# Perceptions and Attitudes Toward Telepsychiatry Among Patients and Psychiatrists: A Mixed-Methods Study in Saudi Arabia

**DOI:** 10.7759/cureus.88309

**Published:** 2025-07-19

**Authors:** Turki M Alanzi, Wejdan Arif, Afrah Alreshidi, Dalal Aldawsari, Omnyah A Sanyour, Basel Alkishi, Alwaleed Alhazzaa, Malath Alsulaihbi, Hayam Alenezi, Firdevs Isa, Jumanah Khayyat, Nouf Alanzi

**Affiliations:** 1 Health Information Management and Technology (HIMT), Imam Abdulrahman Bin Faisal University, Dammam, SAU; 2 Department of Radiological Sciences, King Saud University, Riyadh, SAU; 3 ‏Department of Public Health, College of Public ‏Health, University of Hail, Hail, SAU; 4 ‏College of Medicine, King Saud Bin Abdulaziz University for Health Sciences, Riyadh, SAU; 5 Department of Psychiatry, King Saud Medical City (KSMC), Madinah, SAU; 6 College of Medicine, King Faisal University, Al-ahsa, SAU; 7 Basic Medical Sciences, College of Medicine, King Saud Bin Abdulaziz University for Health Sciences, Riyadh, SAU; 8 Department of Emergency Medicine, King Abdullah Medical Complex, Jeddah, SAU; 9 Department of Medicine, Northern Border University, Arar, SAU; 10 Faculty of Medicine, Umm Al-Qura University, Makkah, SAU; 11 College of Medicine, Umm Al-Qura University, Makkah, SAU; 12 Clinical Laboratory Sciences, Jouf University, Jouf, SAU

**Keywords:** digital mental health, mixed-methods, patient perceptions, psychiatrist attitudes, saudi arabia, telepsychiatry

## Abstract

Background: Telepsychiatry, a branch of telemedicine, has gained prominence in Saudi Arabia as a means of improving access to mental health services. However, comprehensive insights into the perspectives of both patients and providers remain limited.

Aim: This study aimed to explore the perceptions and attitudes of patients and psychiatrists toward telepsychiatry in Saudi Arabia, focusing on its benefits, challenges, and implications for future practice.

Methodology: A mixed-methods design was used, combining quantitative surveys (*n *= 352 patients) and qualitative interviews (*n *= 27 psychiatrists). Statistical analysis included analysis of variance (ANOVA) and t-tests to examine demographic differences, while thematic analysis was applied to interview transcripts.

Results: Patients reported high agreement with telepsychiatry's benefits (*M *= 4.17) and general attitudes (*M *= 4.26), especially regarding convenience and privacy. Age significantly influenced attitudes (*P *< 0.0001) and perceived challenges (*P *< 0.0001), with younger participants being more favorable. Gender differences were also significant for attitudes (*P *= 0.0016) and challenges (*P *= 0.0271). Education level affected technology use and access (*P *< 0.0001). Psychiatrists viewed telepsychiatry as effective for stable cases but expressed concerns over diagnostic accuracy, technical reliability, and confidentiality.

Conclusions: Telepsychiatry is well-received by both patients and providers in Saudi Arabia, especially for non-acute care. To optimize adoption, targeted training, infrastructure investment, and culturally adapted platforms are essential for sustainable integration into hybrid mental healthcare models.

## Introduction

Telepsychiatry, a specialized branch of telemedicine, utilizes telecommunications technologies such as videoconferencing, phone calls, and text messaging to deliver psychiatric care remotely [1-4]. This approach encompasses a broad spectrum of mental health services, including psychiatric evaluations, individual and group therapy, patient education, and medication management, often bridging the gap between psychiatrists and patients separated by distance or mobility constraints [2]. Originally developed to meet the needs of individuals in rural or inaccessible regions, telepsychiatry has rapidly expanded in scope and relevance, particularly in response to the global COVID-19 pandemic, which necessitated a swift transition to remote healthcare delivery [5,6].

The significance of telepsychiatry lies in its potential to enhance access to mental health services, reduce barriers related to geography, stigma, and clinician shortages, and offer flexible, patient-centered care [7-9]. In Saudi Arabia, as in many other countries, telepsychiatry has become increasingly prominent, especially during periods of restricted physical movement and heightened demand for mental health support [10]. Studies have shown that patients generally report high levels of satisfaction with telepsychiatry services, valuing aspects such as comfort, privacy, and convenience [11,12]. Moreover, research suggests that telepsychiatry can be as effective and acceptable as traditional face-to-face care for a variety of psychiatric conditions, with comparable therapeutic outcomes [13,14].

Despite these advantages, several challenges and gaps remain in the implementation and long-term sustainability of telepsychiatry. Digital exclusion, driven by factors such as limited access to devices, internet connectivity, or digital literacy, can hinder equitable access to care, particularly among vulnerable populations [15,16]. Additionally, while the emergency adoption of telepsychiatry during the pandemic demonstrated its feasibility, questions persist regarding its integration into routine clinical practice and its impact on therapeutic relationships, privacy, and patient engagement over time [10].

A critical gap in the literature is the limited understanding of perceptions and attitudes toward telepsychiatry among both patients and psychiatrists in specific cultural and healthcare contexts, such as Saudi Arabia [10]. Existing studies are often restricted by small sample sizes, cross-sectional designs, or a focus on patient satisfaction without parallel exploration of provider perspectives. Furthermore, with the rapid digitalization of the healthcare system as a part of the Vision 2030 initiative [17,18], the evidence base in Saudi Arabia remains sparse, with few comprehensive, mixed-methods investigations that capture the nuanced experiences, expectations, and concerns of both service users and providers [10]. Additionally, barriers such as limited digital literacy, concerns about privacy and confidentiality, and infrastructural constraints have been identified as factors influencing the uptake of telepsychiatry services.​ Given these considerations, there is a pressing need for comprehensive research that delves into the nuanced perspectives of both patients and psychiatrists regarding telepsychiatry in Saudi Arabia.

This study aims to address these gaps by employing a mixed-methods approach to explore the perceptions and attitudes toward telepsychiatry among patients and psychiatrists in Saudi Arabia. By integrating quantitative and qualitative data, the research seeks to provide a holistic understanding of the facilitators and barriers to telepsychiatry adoption, inform future policy and practice, and contribute to the development of culturally sensitive, effective, and sustainable telepsychiatric services in the region.

Background

Telepsychiatry has emerged as a vital component of mental healthcare delivery, especially in response to the COVID-19 pandemic. Numerous studies have explored patient and psychiatrist attitudes toward this modality, revealing a nuanced mix of acceptance, satisfaction, and concerns shaped by access, technological literacy, and clinical suitability.

Patient attitudes toward telepsychiatry have generally been positive, with many reporting high levels of satisfaction and convenience. Studies [19-21] found that patients appreciated the reduction in travel time and associated costs, particularly those in rural areas. Similarly, recent studies [22-24] reported that a majority of patients preferred telepsychiatry over in-person visits due to improved scheduling flexibility. However, concerns remain regarding dissatisfaction with non-verbal feedback, privacy, technological barriers, and the perceived lack of personal connection [23-25]. Psychiatrists have shown cautious optimism. Hubley et al. [26] noted that many psychiatrists initially expressed skepticism, especially regarding diagnostic accuracy and therapeutic rapport, mainly preferred for initial assessments [24,27]. Yet, with increased exposure, attitudes improved as clinicians recognized the efficiency and expanded reach of telepsychiatry. A cross-sectional survey by Smith et al. [28] revealed that over 75% of psychiatrists found telepsychiatry to be an acceptable alternative to face-to-face care in non-acute scenarios, although some continued to express concerns about patient engagement and data security. Similarly, studies have reported that experienced practitioners (>5 teleconsultations) show 2.3× higher acceptance rates than novices, while clinic-based psychiatrists are 1.8× more favorable than hospital-based counterparts [24,27].

The benefits of telepsychiatry are well-documented. Studies [29,30] have shown it increases access to care, particularly for underserved and geographically isolated populations. Cost-effectiveness is another frequently cited benefit, with both patients and health systems reporting financial savings. Clinical outcomes for common conditions such as depression, post-traumatic stress disorder, and schizophrenia are reported to be comparable to those achieved through in-person care [21,24]. Moreover, telepsychiatry facilitates continuity of care during crises, such as pandemics or natural disasters, where physical appointments are disrupted [31,32]. Clinicians also benefit from greater flexibility in scheduling and the ability to conduct home-based assessments, offering insights into patients’ living conditions.

Key challenges about telepsychiatry include digital literacy, internet access, and concerns over confidentiality. Patients lacking familiarity with digital platforms or reliable internet connectivity are often excluded, creating a digital divide [33]. For older adults or individuals with cognitive impairments, these barriers are particularly pronounced. From a provider standpoint, issues such as medico-legal liability, reimbursement policies, and integration into existing workflows pose additional hurdles [34]. Digital literacy gaps affect 22-35% of elderly and low-income patients, while 18% cite privacy concerns as adoption barriers [35]. There is also variability in telepsychiatry regulations across regions, further complicating its widespread adoption.

Patient engagement in telepsychiatry appears to be comparable to, or even higher than, in-person care. Bashshur et al. [36] found that patients with anxiety and depression often feel more comfortable discussing sensitive issues from their own homes. Similarly, the familiarity of the home environment may enhance therapeutic rapport. Satisfaction levels are typically high, with patients highlighting the convenience and privacy of remote sessions. However, technical disruptions and occasional discomfort with video interactions can negatively impact engagement [37]. Accessibility is a critical factor shaping the telepsychiatry experience. Digital literacy, particularly among elderly and low-income populations, remains a significant barrier [38]. Awareness campaigns and user-friendly interfaces are essential to promote equitable access. Some studies suggest that providing pre-session training or technical support improves both confidence and session effectiveness [39].

Looking ahead, telepsychiatry is expected to become a permanent fixture in mental health services. Hybrid models, combining in-person and virtual care, are likely to dominate, offering flexibility while preserving therapeutic quality. Advances in AI and digital platforms may further enhance diagnosis and monitoring. Nonetheless, future developments must address equity concerns, standardization of practices, and robust evidence for long-term outcomes [40].

## Materials and methods

Study settings and participants

This mixed-methods study combined quantitative surveys and qualitative interviews to gain a comprehensive understanding of participants’ perceptions and attitudes. Conducted across multiple healthcare facilities in Saudi Arabia-including both public and private mental health centers offering telepsychiatry services, the study involved two primary groups: patients who had engaged in at least one telepsychiatry session, and licensed psychiatrists currently practicing telepsychiatry. Patients were recruited from outpatient psychiatric clinics and were aged 18 years and older, representing a range of mental health conditions. Psychiatrists were selected based on their direct involvement in delivering psychiatric care via telecommunication platforms. Inclusion criteria for both groups included willingness to participate and the ability to provide informed consent, while individuals with cognitive impairments or language barriers that prevented effective communication were excluded. Eligibility criteria for patients included having participated in at least one telepsychiatry session and being able to complete a survey in Arabic or English. The mixed-methods approach enabled a richer exploration of both statistical trends and in-depth personal experiences across varied demographic and professional backgrounds.

Recruitment and sampling

Participants were recruited using a convenience and purposive sampling strategy [41] to ensure inclusion of individuals with relevant experience in telepsychiatry. For the quantitative phase, only patients were considered, and for the qualitative phase, only psychiatrists were considered. For the quantitative phase, patients were invited to participate through electronic invitations distributed via hospital networks, mental health clinics, and professional psychiatric associations in Saudi Arabia. The sample size for the survey was calculated using Cochran’s formula [42], based on a 95% confidence level, a 5% margin of error, and a maximum variability assumption (50% proportion), resulting in a minimum required sample of 383 respondents.

For the qualitative phase, a subset of survey participants was invited for semi-structured interviews based on their professional background. The sample size of 20-30 psychiatrists is intended to provide a rich dataset while being manageable within the study's timeframe and resources, as observed in similar studies [43-45]. A purposive sampling strategy was employed to select psychiatrists with direct experience in telepsychiatry. Inclusion criteria required participants to be licensed psychiatrists currently practicing in Saudi Arabia, with at least six months of experience providing telepsychiatric care, and able to participate in an interview in either Arabic or English. To ensure diverse perspectives, maximum variation sampling was applied based on work setting (public vs. private), geographic region (urban vs. rural), years of professional experience (early-, mid-, and senior-career), and frequency of telepsychiatry use (occasional vs. regular users). This stratification aimed to capture a wide range of clinical insights and contextual factors influencing attitudes toward telepsychiatry, thereby enhancing the depth and transferability of the qualitative findings.

Questionnaire design

The study employed both a structured questionnaire (Appendix A) for the quantitative phase and a semi-structured interview guide for the qualitative phase to explore perceptions and attitudes toward telepsychiatry among patients and psychiatrists. The quantitative questionnaire was developed based on a review of existing validated tools and literature [19-40] in the fields of telemedicine and mental health. It included demographic questions and Likert-scale items covering five core domains: general attitudes (five items), perceived benefits (five items), perceived challenges (five items), technology use and access (three items), overall experience (three items), and feedback/suggestions (three items). Content validity was ensured through expert review by two professors from the eHealth department, while face validity was assessed through pilot testing with a small sample of 12 patients. Professors were chosen based on their expertise in eHealth and digital mental health research, as well as their extensive experience in survey design and telemedicine evaluation. Both reviewers hold doctoral degrees in health informatics and clinical psychology, respectively, and have published extensively in peer-reviewed journals on topics related to telepsychiatry, digital health interventions, and patient-centered care. Based on the feedback, minor revisions were made to improve clarity and relevance. The questionnaire was administered in both Arabic and English to accommodate participants’ language preferences, with careful translation and back-translation procedures followed to maintain accuracy and consistency. The internal consistency of the questionnaire was assessed using Cronbach’s alpha, which yielded values above 0.70 for all constructs, indicating an acceptable level of reliability [46]. The perception and attitude scores of patients were calculated using responses to a structured questionnaire comprising Likert-scale items across several domains, including general attitudes, perceived benefits, perceived challenges, technology use and access, and overall experience. Each item was rated on a 5-point Likert scale, ranging from 1 (strongly disagree) to 5 (strongly agree). For each participant, domain-specific scores were computed by averaging the responses to items within that domain. The overall perception and attitude scores were then derived by averaging the relevant domain scores, with higher scores indicating more positive perceptions and attitudes toward telepsychiatry. For interpretation, scores closer to 5 reflect strong agreement and a favorable view of telepsychiatry, while scores nearer to 1 indicate strong disagreement and a less favorable attitude.

For the qualitative phase, a semi-structured interview guide was designed to allow for in-depth exploration of individual experiences, attitudes, and contextual factors influencing perceptions of telepsychiatry. The interview guide included open-ended questions focusing on participants' initial expectations, satisfaction with telepsychiatry sessions, perceived effectiveness compared to in-person care, technical or communication barriers encountered, and suggestions for improvement. Additional probes were used to encourage elaboration and explore emerging themes. The interview guides were reviewed by subject matter experts for content relevance and cultural appropriateness, and pilot-tested to ensure clarity and flow. All interviews were conducted in the participants’ preferred language (Arabic or English), recorded with consent, and transcribed verbatim for thematic analysis. This dual design allowed for triangulation of data and a comprehensive understanding of both measurable trends and personal narratives in the adoption of telepsychiatry in Saudi Arabia.

Data collection

Data collection for this mixed-methods study was conducted in two phases: quantitative and qualitative simultaneously from February 25, 2025, to March 25, 2025, across selected healthcare facilities in Saudi Arabia. In the quantitative phase, data were gathered using structured, self-administered questionnaires distributed electronically to improve accessibility. An electronic questionnaire was disseminated through a survey link via email and hospital communication systems. Qualitative data were collected through semi-structured interviews with a purposively selected subset of survey participants. Participants were contacted via phone or email and invited to take part in interviews conducted either in person, by phone, or through secure video conferencing platforms, depending on their preference and convenience. Each interview lasted approximately 30-45 minutes and was conducted in Arabic or English, based on the participant's language preference. All interviews were audio-recorded with informed consent and subsequently transcribed verbatim. Interviewers used a flexible guide to explore themes identified in the survey and probe deeper into individual experiences, challenges, and suggestions related to telepsychiatry.

Data analysis

Data analysis for this mixed-methods study was conducted in two distinct but complementary phases, aligning with the quantitative and qualitative components of the research design. For the quantitative data, responses from the structured questionnaires were coded and entered into the Statistical Package for Social Sciences (SPSS) version 26 (IBM Corp., Armonk, NY). Descriptive statistics, such as means, standard deviations, and frequencies, were calculated to summarize participant demographics and overall responses across the five key domains: general attitudes, perceived benefits, perceived challenges, technology use and access, and overall experience. Inferential statistics, including one-way analysis of variance (ANOVA) and independent samples t-tests, were employed to examine differences in perceptions across demographic variables such as age, gender, and education level. A significance level of *P* < 0.05 was used to determine statistical significance.

For the qualitative phase, thematic analysis was conducted using the six-step approach by Braun and Clarke [47]. Transcribed interviews were first read and then re-read to ensure familiarity with the data. Initial codes were generated systematically, followed by the identification and development of themes that captured the depth and complexity of participant experiences. Coding and theme development were carried out using NVivo software to facilitate the organization and retrieval of qualitative data. Themes were then reviewed, refined, and defined to ensure they accurately represented the data and aligned with the research objectives.

Triangulation of findings from both data sets was performed to enhance the validity and comprehensiveness of the study. Patterns and contradictions between quantitative trends and qualitative narratives were analyzed to provide a nuanced understanding of the perceptions and attitudes toward telepsychiatry. This integrative analysis enabled the study to draw robust conclusions and offer evidence-based recommendations for improving telepsychiatry services in the Saudi Arabian context.

Ethical considerations

Ethical approval for this study was obtained from the appropriate institutional review board in Saudi Arabia. All participants were provided with detailed information about the study and gave written informed consent before participation. Confidentiality was strictly maintained by anonymizing data and securely storing all records. Participation was voluntary, with the option to withdraw at any stage without penalty. Cultural sensitivity and privacy were upheld throughout, particularly during interviews involving mental health topics, ensuring the study adhered to national and international ethical research standards.

## Results

Survey results

Table 1 presents the demographic characteristics of the participants. The majority of respondents were between 18 and 34 years old, with 78 (22.16%) aged 18-24 and 82 (23.30%) aged 25-34, indicating a predominantly younger sample. Participants aged 35-44 comprised 75 individuals (21.31% of the sample), while those aged 45-54 accounted for 48 individuals (13.64%). Older age groups were less represented, with 37 (10.51%) aged 55-64 and 32 (9.09%) aged over 64 years. In terms of gender, females slightly outnumbered males, making up 190 (53.98%) of the participants compared to 162 (46.02%) males. Regarding education levels, a substantial proportion of participants held a Bachelor's degree (138, 39.20%), followed by those with a Master's degree (77, 21.88%), and a Diploma (74, 21.02%). Participants with only primary or secondary education constituted 53 (15.06%) of the sample, while a small minority (10, 2.84%) had attained a Doctoral degree.

**Table 1:** Participants' demographics.

Variables		*n* (%)
Age (in years)	18-24	78 (22.16%)
	25-34	82 (23.3%)
	35-44	75 (21.31%)
	45-54	48 (13.64%)
	55-64	37 (10.51%)
	>64	32 (9.09%)
Gender	Male	162 (46.02%)
	Female	190 (53.98%)
Education	Primary/Secondary education	53 (15.06%)
	Diploma	74 (21.02%)
	Bachelor's degree	138 (39.2%)
	Master's degree	77 (21.88%)
	Doctoral degree	10 (2.84%)

Table 2 summarizes patients’ perceptions and attitudes toward telepsychiatry. Overall, participants reported highly positive general attitudes, with mean scores ranging from 4.15 to 4.42, showing strong comfort with technology and confidence in telepsychiatry as an effective alternative to in-person consultations. Perceived benefits were similarly rated favorably (means between 4.11 and 4.21), reflecting appreciation for convenience, privacy, and reduced stigma, especially for patients in remote areas. Moderate concerns were noted under perceived challenges, with mean scores between 3.56 and 3.74. Participants expressed some worries about technical issues, confidentiality, and reduced personal connection, although these concerns were less strongly rated than the benefits. Regarding technology use and access, participants reported good readiness (mean = 3.86), although a slight preference for face-to-face consultations (mean = 3.77) persisted. Overall experience scores were moderate (means around 3.20-3.23), suggesting general satisfaction but also room for improvement. Feedback indicated that patients supported better technical support and wider integration of telepsychiatry services (mean = 3.18-3.25). In summary, patients showed a positive perception of telepsychiatry, while recognizing certain limitations.

**Table 2:** Patients’ perceptions and attitudes toward telepsychiatry.

Categories	Items	Mean	Standard deviation
General Attitudes	I am comfortable using technology for mental health consultations.	4.42	0.57
	I believe telepsychiatry is an effective alternative to in-person consultations.	4.30	0.54
	I would feel comfortable discussing my mental health issues through telepsychiatry.	4.15	0.59
	I think telepsychiatry improves my access to mental health services.	4.18	0.55
	Telepsychiatry is just as effective as in-person visits for managing my mental health.	4.22	0.56
Perceived Benefits	Telepsychiatry is convenient for me in terms of time and location.	4.20	0.54
	Telepsychiatry offers greater privacy compared to in-person visits.	4.19	0.58
	I believe telepsychiatry helps reduce the stigma associated with seeking mental healthcare.	4.21	0.56
	Telepsychiatry allows me to feel more comfortable and relaxed during my consultation.	4.14	0.54
	Telepsychiatry is a good option for individuals who live in remote areas.	4.11	0.58
Perceived Challenges	I am concerned about the technical issues (e.g., poor internet connection) during telepsychiatry sessions.	3.68	0.68
	I worry about the security and confidentiality of my personal information during telepsychiatry sessions.	3.74	0.67
	I feel that telepsychiatry may not allow for a sufficient level of personal connection with the provider.	3.70	0.65
	I am concerned that telepsychiatry may not address my mental health needs as well as in-person sessions.	3.70	0.67
	I am uncomfortable with the idea of discussing sensitive mental health topics over a video call.	3.56	0.70
Technology use and access	I am confident in my ability to use the technology required for telepsychiatry sessions (e.g., videoconferencing tools).	3.86	0.67
	I have access to the necessary technology (smartphone, internet, etc.) to participate in telepsychiatry sessions.	3.86	0.70
	I prefer face-to-face consultations over telepsychiatry for my mental healthcare.	3.77	0.69
Overall Experience	I would recommend telepsychiatry to others who are seeking mental health support.	3.21	0.77
	I feel that my telepsychiatry session was just as effective as a traditional in-person session.	3.20	0.74
	I am satisfied with the overall quality of my telepsychiatry experience.	3.23	0.79
Feedback/Suggestions	I believe telepsychiatry services could be improved by providing better technical support.	3.25	0.78
	I think that telepsychiatry should be offered alongside in-person options to provide more flexibility.	3.20	0.75
	I feel that telepsychiatry should be integrated more widely into mental health services.	3.18	0.73

**Figure 1:**
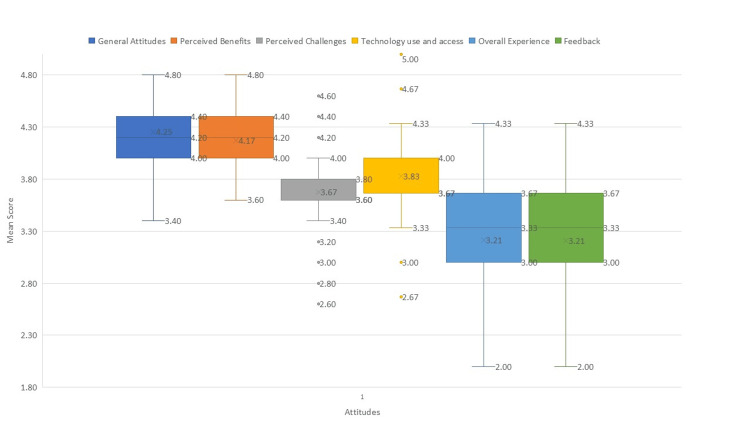
Boxplots of participants’ attitudes and perceptions toward telepsychiatry.

Figure 1 illustrates the distribution of participants’ attitudes and perceptions toward telepsychiatry across six categories. General attitudes and perceived benefits showed the highest median scores (around 4.2), with relatively narrow interquartile ranges, indicating consistently positive responses. Perceived challenges had a lower median (around 3.7), reflecting moderate concerns regarding issues such as technical reliability and personal connection. Technology use and access had a median of approximately 3.8, suggesting good confidence and accessibility, though with slightly greater variability. In contrast, overall experience demonstrated the lowest medians (around 3.2), with wider ranges and several lower outliers, suggesting more mixed satisfaction levels and diverse opinions on service improvements. Overall, the figure highlights that while participants generally held favorable views about telepsychiatry’s benefits and usability, concerns regarding service effectiveness and areas for improvement remained evident.

The ANOVA results presented in Table 3 reveal significant age-related differences in participants' perceptions towards telepsychiatry, specifically in the areas of general attitudes and perceived challenges. For general attitudes, the *P*-value (<0.0001) indicates a statistically significant difference between age groups, with younger participants (aged 18-24) expressing slightly more positive attitudes (mean = 4.32) than older groups, particularly those aged 55-64 (mean = 4.11). In contrast, there were no significant differences observed in perceived benefits (*P* = 0.2631), technology use and access (*P* = 0.6444), or overall experience (*P* = 0.4451), suggesting that age does not significantly affect perceptions in these areas. Regarding perceived challenges, the results again show a statistically significant difference (*P* < 0.0001), with younger participants (18-24, mean = 3.71) reporting fewer challenges compared to older age groups, particularly those aged 55-64 (mean = 3.41), who experienced more challenges. These findings indicate that while attitudes and perceived challenges toward telepsychiatry vary significantly with age, other factors such as perceived benefits, technology use, and overall experience remain relatively consistent across different age groups.

**Table 3:** Differences in participants' perceptions (by age) in relation to their attitudes and perceptions toward telepsychiatry using ANOVA. *Statistically significant difference. ANOVA, analysis of variance

Factors	Age (in years)	n	Mean	Variance	*F*-value	*P*-value
General attitudes	18-24	78	4.32	0.06	5.8841	<0.0001*
	25-34	82	4.33	0.06		
	35-44	75	4.23	0.05		
	45-54	48	4.19	0.07		
	55-64	37	4.11	0.05		
	>64	32	4.21	0.10		
Perceived benefits	18-24	78	4.21	0.07	1.3004	0.2631
	25-34	82	4.16	0.05		
	35-44	75	4.19	0.06		
	45-54	48	4.11	0.06		
	55-64	37	4.14	0.06		
	>64	32	4.20	0.07		
Perceived challenges	18-24	78	3.71	0.06	11.3138	<0.0001*
	25-34	82	3.70	0.09		
	35-44	75	3.74	0.08		
	45-54	48	3.78	0.07		
	55-64	37	3.41	0.10		
	>64	32	3.49	0.12		
Technology use and access	18-24	78	3.81	0.17	0.6726	0.6444
	25-34	82	3.78	0.18		
	35-44	75	3.89	0.10		
	45-54	48	3.79	0.22		
	55-64	37	3.86	0.18		
	>64	32	3.85	0.19		
Overall experience	18-24	78	3.26	0.18	0.9555	0.4451
	25-34	82	3.17	0.17		
	35-44	75	3.20	0.19		
	45-54	48	3.21	0.17		
	55-64	37	3.32	0.26		
	>50	64	3.14	0.22		

The results in Table 4 reveal significant gender-based differences in participants' perceptions toward telepsychiatry in the areas of general attitudes and perceived challenges. For general attitudes, the *P*-value (0.0016) indicates a statistically significant difference, with males reporting slightly more positive attitudes (mean = 4.30) than females (mean = 4.21). In terms of perceived benefits, there is no significant difference between genders, as indicated by the *P*-value (0.9677), with both males and females having identical means (4.17). The results also show a significant difference in perceived challenges (*P* = 0.0271), with males reporting slightly more challenges (mean = 3.71) than females (mean = 3.64). There are no significant gender differences in technology use and access (*P* = 0.1993) or overall experience (*P* = 0.777), as both genders report similar means in these areas. These findings suggest that while general attitudes and perceived challenges differ between genders, perceptions of benefits, technology use, and overall experience are relatively consistent across male and female participants.

**Table 4:** Differences in participants' perceptions (by gender) about their attitudes and perceptions toward telepsychiatry using t-test. *Statistically significant difference.

Factors	Gender	n	Mean	Variance	*t*-value	*P*-value
General attitudes	Male	162	4.30	0.08	3.1457	0.0016*
	Female	190	4.21	0.06		
Perceived benefits	Male	162	4.17	0.06	0.0404	0.9677
	Female	190	4.17	0.06		
Perceived challenges	Male	162	3.71	0.08	2.2510	0.0271*
	Female	190	3.64	0.11		
Technology use and access	Male	162	3.86	0.17	1.2832	0.1993
	Female	190	3.80	0.16		
Overall experience	Male	162	3.21	0.19	0.2831	0.777
	Female	190	3.22	0.19		

The ANOVA results in Table 5 indicate significant differences in participants' perceptions toward telepsychiatry based on education level, specifically in the area of technology use and access. The *P*-value (< 0.0001) for this factor shows a statistically significant difference, with participants holding higher degrees, particularly those with a master's (mean = 4.03) and doctoral degrees (mean = 4.00), reporting better access and use of technology compared to those with primary/secondary education (mean = 3.72). No significant differences were found in general attitudes (*P* = 0.1045), perceived benefits (*P* = 0.4438), perceived challenges (*P *= 0.2961), or overall experience (*P* = 0.2594), suggesting that education level does not significantly affect perceptions in these areas. Although there are some variations in means across different educational groups, these differences are not statistically significant. These findings imply that while education level impacts technology use and access, it does not appear to strongly influence other perceptions towards telepsychiatry.

**Table 5:** Differences in participants' perceptions (by education) in relation to their attitudes and perceptions toward telepsychiatry using ANOVA. *Statistically significant difference. ANOVA, analysis of variance

Factors	Education	n	Mean	Variance	*F*-value	*P*-value
General attitudes	Primary/Secondary education	53	4.23	0.06	1.9325	0.1045
	Diploma	74	4.26	0.07		
	Bachelor's degree	138	4.22	0.07		
	Master's degree	77	4.31	0.06		
	Doctoral degree	10	4.36	0.03		
Perceived benefits	Primary/Secondary education	53	4.17	0.06	0.9347	0.4438
	Diploma	74	4.19	0.06		
	Bachelor's degree	138	4.18	0.06		
	Master's degree	77	4.13	0.06		
	Doctoral degree	10	4.26	0.07		
Perceived challenges	Primary/Secondary education	53	3.62	0.11	1.2339	0.2961
	Diploma	74	3.71	0.07		
	Bachelor's degree	138	3.66	0.09		
	Master's degree	77	3.70	0.12		
	Doctoral degree	10	3.78	0.02		
Technology use and access	Primary/Secondary education	53	3.72	0.18	7.727	<0.0001*
	Diploma	74	3.82	0.16		
	Bachelor's degree	138	3.75	0.15		
	Master's degree	77	4.03	0.15		
	Doctoral degree	10	4.00	0.10		
Overall experience	Primary/ Secondary education	53	3.25	0.13	1.3269	0.2594
	Diploma	74	3.28	0.20		
	Bachelor's degree	138	3.18	0.17		
	Master's degree	77	3.16	0.24		
	Doctoral degree	10	3.37	0.31		

Interview results

General Views on Telepsychiatry

Most psychiatrists (22/27, 81.48%) expressed a positive overall perception of telepsychiatry, describing it as a valuable and practical addition to mental healthcare delivery. Many (19/27, 70.37%) felt that telepsychiatry was moderately to highly effective compared to in-person consultations, although a few (8/27, 29.62%) stated that its effectiveness depended heavily on the specific case and the patient's condition.

The main advantages cited included improved accessibility for remote and underserved areas (23/27, 85.18%), greater convenience for both patients and clinicians (18/27, 66.67%), and reduced appointment waiting times (15/27, 55.55%). However, only some (11/27, 40.74%) interviewees believed that telepsychiatry could offer the same quality of care as face-to-face consultations, primarily for follow-up and stable cases. Complex diagnostic assessments were still perceived as better suited for in-person visits.

Regarding accessibility, most psychiatrists (24/27, 88.89%) agreed that telepsychiatry significantly enhanced service reach for patients in remote regions, often being the only viable option for some communities.

Challenges and Concerns

The most frequently reported challenge was technical issues, particularly unreliable internet connections, cited by many (20/27, 74.07%) psychiatrists. In addition, more than half (16/27, 59.25%) voiced concerns about reduced diagnostic accuracy when relying solely on virtual interactions, especially for complex psychiatric cases.

To address technical problems, about half (14/27, 51.85%) of the interviewees indicated they had contingency plans in place, such as switching to audio-only calls or rescheduling sessions. However, the disruption was acknowledged to affect the flow and quality of care.

When asked about unsuitability, most psychiatrists (21/27, 77.78%) stated that certain conditions-such as severe psychosis, suicidality, and cognitive impairments-were not ideal for telepsychiatry. On confidentiality, many (17/27, 62.96%) participants expressed concerns, particularly when patients connected from non-private locations. Measures such as secure platforms and patient education on privacy practices were commonly employed, although complete assurance was difficult.

Patient Engagement and Satisfaction

Regarding patient perceptions, most psychiatrists (20/27, 74.07%) believed that patients generally responded positively to telepsychiatry, valuing its convenience. However, a few (7/27, 25.92%) noted that some patients, particularly older adults, were initially hesitant or uncomfortable.

On the therapeutic relationship, many psychiatrists (18/27, 66.67%) felt that building rapport was somewhat more challenging via telepsychiatry, especially during initial consultations. Nevertheless, some (9/27, 33.34%) believed that once familiarity was established, the quality of the therapeutic relationship was comparable to that of in-person sessions.

When considering patient engagement, many (16/27, 59.25%) interviewees observed that participation rates remained similar or even improved slightly, whereas some (11/27, 40.74%) noted occasional difficulties in maintaining patient focus during virtual sessions.

Regarding stigma reduction, many psychiatrists (19/27, 70.37%) agreed that telepsychiatry helped lessen stigma, as patients could seek care more privately and discreetly from their homes.

Technology and Training

Comfort with technology was high, with most psychiatrists (21/27, 77.78%) reporting they were comfortable or very comfortable using telepsychiatry platforms. However, a few (6/27, 22.22%) indicated a need for more technical support, particularly during the early phases of adoption.

To improve practice, many psychiatrists (18/27, 66.67%) recommended more structured training sessions focused on both technology use and adapting clinical skills for virtual settings.

Regarding platform adequacy, many psychiatrists (17/27, 62.96%) found the platforms generally sufficient, while some (10/27) highlighted the need for more specialized features, such as mental health-specific assessment tools and enhanced cybersecurity measures.

Looking forward, most psychiatrists (23/27, 85.18%) believed telepsychiatry could be integrated more widely, provided there was continuous investment in infrastructure and professional development.

Future Outlook

When asked about the future, most psychiatrists (24/27, 88.89%) envisioned telepsychiatry becoming a permanent and increasingly important part of psychiatric practice, particularly within hybrid care models. Finally, nearly all (25/27, 92.59%) interviewees said they would recommend telepsychiatry to colleagues, emphasizing its role in bridging gaps in mental health service delivery, while recognizing that it may not be suitable for every case.

## Discussion

This study aimed to explore the perceptions and attitudes toward telepsychiatry among patients and psychiatrists in Saudi Arabia, using a mixed-methods approach. Overall, the findings affirm that both groups view telepsychiatry positively, while simultaneously highlighting key challenges related to technological infrastructure, patient engagement, and clinical appropriateness-particularly in complex cases. These results are largely consistent with prior research, while also contributing novel insights specific to the Saudi Arabian context.

In line with previous studies [19-21], patients in this study expressed favorable attitudes toward telepsychiatry, appreciating its convenience, privacy, and ability to reduce stigma. This supports the conclusions drawn by Bashshur et al. [36], who noted that patients often feel more at ease discussing mental health concerns from the comfort of their homes. Similarly, participants in the current study identified increased access, especially in remote areas, as a major benefit-echoing findings from prior research [29,30] which emphasized telepsychiatry's capacity to bridge geographic gaps in mental healthcare delivery.

Despite high satisfaction levels, participants in both patient and psychiatrist groups raised concerns about reduced non-verbal communication, difficulties in establishing therapeutic rapport, and potential technical issues. These observations parallel concerns in the literature regarding the limitations of virtual consultations, particularly for initial assessments and severe psychiatric conditions [23-25, 26, 27]. The study further validates the view of Smith et al. [28], who reported that psychiatrists view telepsychiatry as an acceptable alternative in non-acute scenarios, but with reservations for more complex clinical interactions.

Significantly, the study uncovered age-related disparities in perceptions. Younger patients reported more favorable general attitudes and fewer perceived challenges compared to older adults, consistent with previous findings suggesting that digital literacy varies significantly with age [35,38]. As digital exclusion remains a major barrier, especially for the elderly and those with limited technological access, the Saudi mental health system must address these gaps through targeted digital literacy initiatives and user-friendly platforms.

Education was another key variable influencing perceptions, particularly about technology use and access. Participants with higher education levels reported greater confidence and readiness in using telepsychiatry tools-echoing studies [33,38] that identify digital literacy as a predictor of telepsychiatry uptake. These findings underscore the need for national-level efforts to standardize training and ensure equitable access across educational backgrounds.

From the provider perspective, while most psychiatrists recognized telepsychiatry's benefits, concerns remained regarding diagnostic accuracy, privacy, and platform limitations. This aligns with previous studies [26,28,34], which highlight medico-legal concerns and the need for platforms better tailored to psychiatric assessment. Notably, the current findings expand on this by showing that psychiatrists in Saudi Arabia largely support the integration of telepsychiatry within hybrid models-suggesting a practical pathway forward that blends in-person care with virtual consultations, as advocated in the literature [40].

Recommendations for the Saudi context include the expansion of telepsychiatry infrastructure, especially in underserved regions, and the establishment of national guidelines that address confidentiality, clinical suitability, and quality assurance. Additionally, structured training programs for both providers and patients are essential to enhance confidence in using digital platforms, as many psychiatrists in this study recommended. Enhancing platforms with specialized mental health features and strengthening cybersecurity protocols would further boost acceptance and reliability.

While the findings mirror global trends, they also highlight specific challenges and opportunities unique to the Saudi healthcare landscape. As part of Saudi Arabia’s Vision 2030, which emphasizes digital health transformation [17,18], integrating telepsychiatry more comprehensively could significantly improve mental health service delivery-provided that technological, educational, and regulatory challenges are addressed. Future studies should explore longitudinal outcomes and evaluate telepsychiatry’s impact on clinical effectiveness across diverse psychiatric populations within the Kingdom.

Implications and limitations

The study enriches theoretical frameworks on technology adoption in healthcare by demonstrating how cultural factors and infrastructural realities influence the acceptance of telepsychiatry, particularly in non-Western settings. It validates core components of models like the Unified Theory of Acceptance and Use of Technology while highlighting the need to integrate digital literacy and sociocultural competence as critical dimensions. The findings also underscore the importance of hybrid care models in bridging gaps between patient expectations and clinician capabilities, offering a new lens for understanding how blended care approaches can enhance therapeutic alliances in digital mental health services. Practically, the study provides a roadmap for Saudi Arabia’s healthcare modernization efforts under Vision 2030, emphasizing the need for targeted clinician training programs, standardized Arabic-language platforms, and public awareness campaigns to address digital hesitancy. The identification of privacy concerns and infrastructural barriers calls for immediate policy interventions, such as secure cloud-based solutions and incentives for clinics adopting hybrid care models, to ensure equitable access and sustained engagement.

While this study offers valuable insights into perceptions of telepsychiatry in Saudi Arabia through a robust mixed-methods approach, several limitations should be acknowledged. First, the sample is skewed toward younger, more educated participants, which may limit the generalizability of findings to older adults or those with lower levels of education who may have differing attitudes or access to telepsychiatry services. This demographic imbalance reflects the nature of electronic recruitment, which inherently favors individuals with greater digital access and literacy, potentially excluding underrepresented or digitally marginalized groups. Second, although the statistical analyses employed (e.g., ANOVA, t-tests) were appropriate for the study design, we did not report effect size measures or conduct multivariate analyses, which could have provided additional depth to the interpretation of demographic influences and interaction effects. Future research should incorporate these methods to better assess the magnitude and interplay of variables influencing perceptions. Finally, as with any cross-sectional design, causal inferences cannot be established, and findings reflect participants’ perceptions at a single point in time. Longitudinal studies are recommended to examine how attitudes toward telepsychiatry evolve with increased exposure and technological advancement.

## Conclusions

In conclusion, this mixed-methods study provides a comprehensive and contextually relevant understanding of the perceptions and attitudes toward telepsychiatry among patients and psychiatrists in Saudi Arabia. The findings reveal high levels of satisfaction and acceptance among patients, particularly regarding convenience, privacy, and improved access, which are in line with previous international and local studies. However, notable challenges persist, including gaps in digital literacy, technological barriers, and concerns about the quality of therapeutic relationships - issues that are also echoed in the broader literature but appear more pronounced in the Saudi context due to cultural and infrastructural factors. Psychiatrists, while generally positive about the potential of telepsychiatry, demonstrate significant variability in technical knowledge and confidence, underscoring the need for targeted training and institutional support. To maximize the benefits of telepsychiatry and ensure equitable, sustainable integration into mental health services, it is recommended that Saudi Arabia prioritize digital literacy initiatives, develop culturally sensitive training programs for clinicians, invest in robust and user-friendly technological infrastructure, and implement clear regulatory frameworks. These steps will help bridge existing gaps, enhance patient and provider engagement, and support the ongoing digital transformation of mental healthcare in alignment with the country’s Vision 2030 goals.

## Appendices

Appendix A: Survey questionnaire: perceptions and attitudes toward telepsychiatry among patients

Section 1: Demographic Information

Age:

18-24

25-34

35-44

45-54

55-64

65 and above

Gender:

Male

Female

Other

Prefer not to say

Highest Level of Education:

High school or below

Some college/university

Bachelor's degree

Master's degree

Doctoral degree

Other (please specify): ________________

Have you used telepsychiatry services before?

Yes

No

Section 2: General Attitudes Toward Telepsychiatry

1.      I am comfortable using technology for mental health consultations.

Strongly Disagree

Disagree

Neutral

Agree

Strongly Agree

I believe telepsychiatry is an effective alternative to in-person consultations.

Strongly Disagree

Disagree

Neutral

Agree

Strongly Agree

I would feel comfortable discussing my mental health issues through telepsychiatry.

Strongly Disagree

Disagree

Neutral

Agree

Strongly Agree

I think telepsychiatry improves my access to mental health services.

Strongly Disagree

Disagree

Neutral

Agree

Strongly Agree

Telepsychiatry is just as effective as in-person visits for managing my mental health.

Strongly Disagree

Disagree

Neutral

Agree

Strongly Agree

Section 3: Perceived Benefits of Telepsychiatry

6.      Telepsychiatry is convenient for me in terms of time and location.

Strongly Disagree

Disagree

Neutral

Agree

Strongly Agree

Telepsychiatry offers greater privacy compared to in-person visits.

Strongly Disagree

Disagree

Neutral

Agree

Strongly Agree

I believe telepsychiatry helps reduce the stigma associated with seeking mental health care.

Strongly Disagree

Disagree

Neutral

Agree

Strongly Agree

Telepsychiatry allows me to feel more comfortable and relaxed during my consultation.

Strongly Disagree

Disagree

Neutral

Agree

Strongly Agree

Telepsychiatry is a good option for individuals who live in remote areas.

Strongly Disagree

Disagree

Neutral

Agree

Strongly Agree

Section 4: Perceived Challenges and Concerns

11.  I am concerned about the technical issues (e.g., poor internet connection) during telepsychiatry sessions.

Strongly Disagree

Disagree

Neutral

Agree

Strongly Agree

I worry about the security and confidentiality of my personal information during telepsychiatry sessions.

Strongly Disagree

Disagree

Neutral

Agree

Strongly Agree

I feel that telepsychiatry may not allow for a sufficient level of personal connection with the provider.

Strongly Disagree

Disagree

Neutral

Agree

Strongly Agree

I am concerned that telepsychiatry may not address my mental health needs as well as in-person sessions.

Strongly Disagree

Disagree

Neutral

Agree

Strongly Agree

I am uncomfortable with the idea of discussing sensitive mental health topics over a video call.

Strongly Disagree

Disagree

Neutral

Agree

Strongly Agree

Section 5: Technology Use and Access

16.  I am confident in my ability to use the technology required for telepsychiatry sessions (e.g., video conferencing tools).

Strongly Disagree

Disagree

Neutral

Agree

Strongly Agree

I have access to the necessary technology (smartphone, internet, etc.) to participate in telepsychiatry sessions.

Strongly Disagree

Disagree

Neutral

Agree

Strongly Agree

I prefer face-to-face consultations over telepsychiatry for my mental health care.

Strongly Disagree

Disagree

Neutral

Agree

Strongly Agree

Section 6: Overall Experience

19.  I would recommend telepsychiatry to others who are seeking mental health support.

Strongly Disagree

Disagree

Neutral

Agree

Strongly Agree

I feel that my telepsychiatry session was just as effective as a traditional in-person session.

Strongly Disagree

Disagree

Neutral

Agree

Strongly Agree

I am satisfied with the overall quality of my telepsychiatry experience.

Strongly Disagree

Disagree

Neutral

Agree

Strongly Agree

Section 7: Suggestions and Feedback

22.  I believe telepsychiatry services could be improved by providing better technical support.

Strongly Disagree

Disagree

Neutral

Agree

Strongly Agree

I think that telepsychiatry should be offered alongside in-person options to provide more flexibility.

Strongly Disagree

Disagree

Neutral

Agree

Strongly Agree

I feel that telepsychiatry should be integrated more widely into mental health services.

Strongly Disagree

Disagree

Neutral

Agree

Strongly Agree

Interview questionnaire for Psychiatrists

Section 1: General Views on Telepsychiatry

What is your overall perception of telepsychiatry as a mode of delivering mental health care?

In your experience, how effective do you think telepsychiatry is compared to in-person psychiatric consultations?

What are the main advantages of using telepsychiatry for mental health care delivery?

Do you believe telepsychiatry can offer the same quality of care as face-to-face consultations? Why or why not?

What do you think about the accessibility of telepsychiatry for patients who live in remote or underserved areas?

Section 2: Challenges and Concerns

What are the primary challenges you face when conducting telepsychiatry sessions?

Do you have any concerns about the effectiveness of diagnosing and treating mental health disorders remotely?

How do you address potential technical issues (e.g., internet connection, platform malfunctions) during a telepsychiatry session?

Are there specific types of mental health issues or conditions that you believe are not suitable for telepsychiatry?

How do you ensure patient confidentiality and privacy during a telepsychiatry session, and do you have concerns in this area?

Section 3: Patient Engagement and Satisfaction

How do you think patients perceive telepsychiatry compared to in-person consultations?

What impact do you believe telepsychiatry has on building a therapeutic relationship with patients?

Have you observed any differences in patient engagement or participation during telepsychiatry sessions compared to in-person visits?

Do you think telepsychiatry can help reduce the stigma associated with seeking mental health care? Why or why not?

Section 4: Technology and Training

How comfortable are you with using the technology required for telepsychiatry sessions?

What types of training or support would be helpful to improve your telepsychiatry practice?

Do you feel that the telepsychiatry platforms you use are adequate for conducting effective mental health care?

Do you think telepsychiatry has the potential to be integrated more widely into psychiatric practice, and what factors would contribute to or hinder this?

Section 5: Future Outlook

How do you see the role of telepsychiatry evolving in the next 5-10 years?

Would you recommend telepsychiatry to other mental health professionals? Why or why not?
